# Hydrocolloid-Based Coatings with Nanoparticles and Transglutaminase Crosslinker as Innovative Strategy to Produce Healthier Fried Kobbah

**DOI:** 10.3390/foods9060698

**Published:** 2020-06-01

**Authors:** Asmaa Al-Asmar, Concetta Valeria L. Giosafatto, Mohammed Sabbah, Loredana Mariniello

**Affiliations:** 1 Department of Chemical Sciences, University of Naples “Federico II”, 80126 Naples, Italy; a.alasmar@najah.edu (A.A.-A.); giosafat@unina.it (C.V.L.G.); 2 Analysis, Poison Control and Calibration Center (APCC), An-Najah National University, P.O. Box 7 Nablus, Palestine; 3 Department of Nutrition and Food Technology, An-Najah National University, P.O. Box 7 Nablus, Palestine; m.sabbah@najah.edu

**Keywords:** acrylamide, kobbah, transglutaminase, pectin, chitosan-nanoparticles, coatings, mesoporous silica nanoparticles, grass pea, HPLC-RP

## Abstract

This study addresses the effect of coating solutions on fried kobbah. Coating solutions were made of pectin (PEC) and grass pea flour (GPF), treated or not with transglutaminase (TGase) and nanoparticles (NPs)—namely mesoporous silica NPs (MSN) or chitosan NPs (CH–NPs). Acrylamide content (ACR), water, oil content and color of uncoated (control) and coated kobbah were investigated. Zeta potential, Z-average and in vitro digestion experiments were carried out. Zeta potential of CH–NPs was stable from pH 2.0 to pH 6.0 around + 35 mV but decreasing at pH > 6.0. However, the Z-average of CH–NPs increased by increasing the pH. All coating solutions were prepared at pH 6.0. ACR of the coated kobbah with TGase-treated GPF in the presence nanoparticles (MSN or CH–NPs) was reduced by 41.0% and 47.5%, respectively. However, the PEC containing CH–NPs showed the higher reduction of the ACR by 78.0%. Water content was higher in kobbah coated by PEC + CH–NPs solutions, while the oil content was lower. The color analysis indicated that kobbah with lower browning index containing lower ACR. Finally, in vitro digestion studies of both coating solutions and coated kobbah, demonstrated that the coating solutions and kobbah made by means of TGase or nanoparticles were efficiently digested.

## 1. Introduction

Kibbeh, kibbe, kobbah (also kubbeh, kubbah, kubbi) (pronunciation varies with region) is Eastern dish made of a ground bulgur (wheat-based food) mixed with minced beef meat formed as balls stuffed with cooked ground meat, onions, nuts and spices. They are usually cooked by deep frying for 8–10 min at high temperatures (160–180 °C), thus they have rough crust and thoroughly browned. They are home-made and consumed fresh or they are sold frozen in the super-markets and consumers can fry them at home [1].

Hydrocolloid materials are used for food protection, as well as for separating the different part of a food [2,3]. Coatings represent a thin layer of edible molecules that are laid on the surface of a food product and can be used to protect high perishable aliments. Pectin (PEC) is a polysaccharide present in the plant cell wall containing mainly galacturonic acid, but highly variable in composition, structure and molecular weight [4]. PEC is known as food additive (E440), useful for thickening mainly jam and marmalades and other products, since is provided with gelling properties [5]. Yossef [6], found that strawberry fruits dipped in PEC-based solutions retained physico-chemical properties as the same fruit protected by other hydrocolloid molecules, such as soy proteins, gluten or starch. Moreover, protein-based such as grass pea flour (GPF) was used for its high content in proteins. Grass pea (*Lathyrus sativus* L.) belongs to the leguminous family and is quite important in many Asian and African countries where it is cultivated either for animal feeding or human use. It is characterized by resistance to both abiotic and biotic stresses [7]. GPF containing proteins were able to act as transglutaminase substrates, giving arise to novel bioplastics endowed with improved technological properties than the ones cast without the enzyme [8,9,10].

Microbial transglutaminase (TGase) belongs to a family of enzymes (E.C. 2.3.2.13) (widely distributed in nature from microbes to animals and plants) capable of catalyzing iso–peptides bonds between endo-glutamine and endo-lysine residues belonging to proteins of different nature, giving arises to intra- and inter-molecular crosslinks [2,11]. TGase is widely used in the food industry as technological aid. Recently, Sabbah et al. [12], demonstrated that the proteins of *Nigella sativa* defatted cakes are act as TGase substrates and are responsible to enhance physico-chemical properties of the obtained films.

Using nanoparticles for developing of nanocomposite coatings is a way to improve their features and is of interest also for producing active packaging [13]. Mesoporous silica nanoparticles MSNs (Type MCM-41) are a kind of SiO_2_–based nanoparticles that are promising materials for application in numerous aspects of biomedical and food purposes [14,15,16]. Recently, Fernandez-Bats et al. [17]; Giosafatto et al. [18]; Al-Asmar et al. [3], prepared and characterized the active protein, pectin and chitosan edible films grafted with MSNs, and they concluded that MSNs significantly influence the mechanical and permeability properties of the obtained materials. SiO₂ nanoparticles of different composition are labeled as E551, E554, E556 or E559 and used for instance as an anti-caking agent. The amount ingested daily is estimated to be 1.8 mg/kg (around 126 mg/day for a 70 kg person) [19]. Moreover, McCarthy [20] observed that SiO₂- based NPs with the size of 150 nm and 500 nm do not perform toxic effects on Calu-3 cells.

Chitosan nanoparticles (CH–NPs) are natural materials obtained from the marine byproducts, endowed with not able physico-chemical and antimicrobial characteristics, besides being sustainable and harmless for human health [21,22]. These properties suggest that CH–NPs can be used also as carrier for drug delivery. Lorevice et al. [23], obtained higher mechanical properties by adding CH–NPs to PEC films compared with control, allowing these novel materials to be an alternative to traditional food packaging production. Moreover, addition of small fractions of CH–NPs enhance mechanical and thermal stability of banana puree-based films [24]. Application of CH in foods is gaining interest, specifically after that shrimp chitin-derived CH has been recognized as Generally Recognized as Safe (GRAS) for common use in foods by the US Food and Drug Administration in 2011 [25,26].

Acrylamide (ACR) is a chemical that was discovered in foods in 2002 and it is present in a range of popular foods [27]. ACR is not present in raw foods, but it is formed from natural precursors during food treatment at high-temperature (>120 °C) following Maillard reaction [28]. ACR is formed during frying, baking roasting and toasting of the carbohydrate rich food and cereal products, as well as coffee. In particular, ACR occurs because of the reaction between the free amino acid asparagine and a carbonyl-containing compound. [29]. European Food Safety Authority (EFSA) scientists classified ACR is a ‘probably carcinogenic to humans [30]. Hydrocolloid-based coatings recently become one of several strategies used to mitigate ACR formation and to improve the eating quality of different fried foods such as potato chips [31], bread [32] and fried banana [33]. Very recently, our research group demonstrated the effectiveness of hydrocolloid-based coatings prepared in the presence of TGase in reducing ACR content of French fries [34] and fried falafel, a typical Easter food [35]. In 2004, Al-Dmoor et al. [1] determined the ACR content in fried kobbah (food mostly eaten in Jordan, but also very popular in Palestine) finding values ranging from 2900 to 5300 μg kg^−1^. Thus, kobbah contain ACR in values that impose attention to protect the health of consumers.

The aim of this study was to investigate the influence of different hydrocolloid-based coatings (containing PEC and GPF prepared in the presence or absence of MNS and/or TGase) on ACR, water content, oil content, digestibility and color of fried kobbah. The physicochemical properties of different coating solutions were also evaluated.

## 2. Materials and Methods

### 2.1. Materials

Methanol and ACR standard ≥99.8%, were purchased from Sigma–Aldrich Chemical Company (St. Louis, MO, USA). Acetonitrile HPLC analytical grade, n-hexane and formic acid were obtained from Carlo Erba reagents S.r.l. (Cornaredo, Milan, Italy). Oasis HLB 200 mg, 6 mL solid phase extraction (SPE) cartridges were from Waters (Milford, MA, USA). Syringe filters (0.45- and 0.22-µm PVDF) were from Alltech Associates (Deerfield, Italy). PEC of a low-methylated citrus peel (7%) (Aglupectin USP) was purchased from Silva Extracts s.r.l. (Gorle, Bergamo, Italy) and Activa^®^WM *Streptoverticillium* TGase was supplied by Ajinomoto Co (Tokyo, Japan). Sodium tripolyphosphate (TPP) and glycerol (GLY) was from Merck Chemical Company (Darmstadt, Germany). Chitosan (CH, mean molar mass of 3.7–104 g/mol) was procured from Professor R. Muzzarelli (University of Ancona, Ancona, Italy), with a degree of 9.0% N-acetylation. Grass pea seeds, corn oil, ground bulgur (wheat-based food), minced beef meat, onion, salt and spices were obtained from a local market in Naples (Italy).

### 2.2. Nanoparticles Preparation

MSN (MCM-41) were synthesized and characterized, as described in Fernandez-Bats et al. [17]. However, CH–NPs was prepared by using the ionic gelation method according to Chang et al. [36]. Briefly, adding the TPP 0.5% (*w/v*) dropwise to the CH solution (0.8%) by 40 min stirring the obtained suspension was centrifuged at 22,098× *g* for 10 min at 4 °C, then rinsed three times by Milli-Q water and dry at room temperature.

### 2.3. Kobbah Formulation and Manufacturing

Kobbah was made as described by Brazil et al. [37], soaking ground bulgur flour into hot water (80 °C) for 1 h, then the wheat flour, oil, salt and spices were added and mixed with the soaked ground bulgur. The dough was kept at the refrigerator for 1 h. Stuffing: onions, salt and spices were mixed with minced beef meat then cooked with olive oil. The dough was shaped into balls stuffed with cooked ground meat.

### 2.4. Preparation of the Coating Solutions

GPF was obtained according to Giosafatto et al. [8] and Al-Asmar et al. [34], in particular, the seeds were milled by a laboratory blender LB 20ES (Waring Commercial, Torrington, CT, USA) and the obtained flour was treated with a 425-μm stainless steel sieve (Octagon Digital Endecotts Limited, London, UK). A total of 83 g of GPF (containing 24% *w/w* proteins) were dissolved in 1 L Milli-Q water and the solutions were shacked for 1 h. the pH was brought to 9.0 with 1-M NaOH followed by centrifugation at 12,096× *g* for 10 min. After centrifugation, the supernatant was collected and used to prepare the GPF dipping solution. Nanoparticles, either MSN or CH–NP (1% *w/w* GPF proteins) were added to GPF at pH 6.0 than the solutions were mixed for 30 min at room temperature (GPF; GPF + MSN; GP + CH–NP). TGase (33 U/g of GPF proteins) was used to prepare the GPF + TGase; GPF + MSN + TGase and GPF + CH–NP + TGase, GLY was used as plasticizer (8% *w/w* GPF proteins) in all the GPF coating solutions, then incubated for 2 h at 37 °C. PEC-based solutions (1% *w/v*) prepared as described in Esposito et al. [38], were made from a PEC stock solution (2% *w/v*), then diluted with Milli-Q water. MSN or CH–NP (1% *w/w* PEC) were added to PEC and mixed for 30 min at room temperature (PEC; PEC + MSN; PEC + CH–NP). Each dipping solutions were adjusted to pH 6.0, then was used to coat kobbah before trying.

### 2.5. Dipping and Frying Method

Two hundred grams of kobbah (divided in 5 pieces) were immersed for 30 s into either in H_2_O_d_ (“control” sample) or in one of these coating solutions: (1) GPF; (2) GPF reinforced with MSN (GPF + MSN); (3) GPF reinforced with CH–NP (GPF + CH–NP); (4) TGase-treated (GPF + TGase); (5) TGase-treated reinforced with MSN (GPF + MSN + TGase); (6) TGase-treated reinforced with CH–NP (GPF + CH–NP + TGase); (7) PEC; (8) PEC reinforced with MSN (PEC + MSN); and (9) PEC reinforced with CH–NP (PEC + CH–NP). Moreover, each sample was dripped for 2 min before frying to get rid of the excess of solutions. The frying conditions consisted in 2 L corn oil preheated (using a controlled temperature deep-fryer apparatus (GIRMI, Viterbo, Italy)) to the processing temperature (190 ± 5 °C), then the kobbah were deep fried for 4.5 min. The oil was replaced by new one for each different coating solutions. After frying, kobbah were drained for 2 min to remove oil excess [34,35,39].

### 2.6. Zeta Potential and Z-Average of Coating Solutions

The Zeta potential and particle size (Z-average) of the CH–NP solution (1 mg/mL) prepared at pH 2.0 were obtained by titration from pH 2.0 to pH 7.0, by means of Zetasizer Nano-ZSP (Malvern^®^, Worcestershire, UK) equipped with a He–Ne laser. All coating solutions used in this experiment were also tested for their Zeta potential and Z-average.

### 2.7. Oil and Water Content

The oil content was performed following frying and cooling of each processed samples around (3–5 g) in triplicate. The result was reported as a percentage on dry matter weight by n-hexane solvent extraction using the Soxhlet method [40].

Fried kobbah water content was obtained following the gravimetric method [41] in triplicate.

### 2.8. Acrylamide Detection

#### 2.8.1. Preparation of Acrylamide Standard

ACR standard stock solution (1.0 mg/mL) was obtained as described in Al-Asmar et al. [34,35]. In particular 10 mg of the ACR standard were dissolved in 10 mL of Milli-Q water. From the stock solution, different concentrations of calibration standards (100, 250, 500, 1000, 2000, 3000, 4000 and 5000 µg/L), were prepared, respectively. All series of standard solutions were kept in glass dark bottles at 4 °C until used.

#### 2.8.2. Acrylamide Extraction

ACR extraction was performed as described in Al-Asmar et al. [34,35]. Briefly, about 200 g of fried kobbah, were put in n-hexane for 30 min to get rid of the oil [42]. After that, n-hexane was let to evaporate under fume hood at room temperature, then samples were subjected for ACR extraction. The treated fried kobbah samples were milled at 1300 rpm for 1 min by means of a rotary mill Grindomix GM200, (Retsch GmbH, Haan, Germany). Freeze drying was used to dry the samples before ACR extraction that was carried out by following the procedure of Wang et al. [43]. Briefly, two different tubes were set up for each sample, one for detecting ACR formed in kobbah samples, and the second one to carry out the “Recovery test for ACR in all kobbah types (in each sample 150 μg/L of ACR standard were added”. In both tubes, 1.0 g (dry weight) of sample, was placed in both tubes and only in the second one there was the ACR standard added. Carrez reagent potassium salt and Carrez reagent zinc salts (50 µL) were included in each sample. In each tube, 10 mL of HPLC water were finally added. The samples were put in an incubated shaker for 30 min at 25 °C and 170 rpm, then centrifuged at 7741× *g* for 10 min at 4 °C. The supernatant was filtered with 0.45-µm syringe filter for the clean-up of the Oasis HLB SPE cartridges. The SPE cartridge was preventively conditioned with 2.0 mL of methanol followed by washing with 2 mL of water before loading 2.0 mL of the filtered supernatant, the first 0.5 mL was discarded and the remaining elute collected (~1.5 mL; exact volume was measured by weight and converted by means of density). All extracts were kept in dark glass vials at 4 °C before analysis. The clean sample extracts were further filtered through 0.2-µm nylon syringe filters before HPLC-UV (ultra violet) analysis of fried kobbah [34]. Each determination was performed in triplicate.

#### 2.8.3. HPLC-UV Analysis

HPLC-UV analysis was used to determine the ACR, by using the RP-HPLC method on Beckman Gold HPLC instrument equipped with a dual pump and a diode array detector [34]. The column Synergi™ 4-µm Hydro-RP 80 Å HPLC Column 250 × 3 mm (Phenomenex, Torrance, CA, USA) was used [44].

The operating conditions described also in Al-Asmar et al. [34] were the following: the wavelength detection was 210 nm, a gradient elution of 0.1% formic acid (*v/v*) in water: acetonitrile (97:3, *v/v*) was used. Solvent A was water and Solvent B was acetonitrile, both solvents containing 0.10% (*v/v*) formic acid; flow rate, 1 mL/min. The gradient elution program was applied as follows: 97% A (3% B) for 10 min, increased to 20% A (80% B) from 10 to 12 min and kept at 20% A (80% B) for 5.0 min, increased to 95% B (5% A) from 17 to 19 min and kept at 95% B for 5 min, increased to 97% A (3% B) from 24 to 26 min and kept for 4 min. The injection volume was equal to 20 µL. The total chromatographic runtime was 30 min for each sample and the temperature was kept at 30 °C (GECKO 2000 “HPLC column heater”, Spectra Lab Scientific, Inc., Markham, ON, Canada) to ensure optimal separation. In all samples (ACR standard and fried kobbah-derived), the ACR retention time was 4.9 min.

### 2.9. Color Analysis

Color measurement of food products was considered as an indirect measure of other quality features such as flavor and contents of pigments [45]. Chroma Meter Konica Minolta CR-400 (Japan) was utilized to determine L*, a*, b* values of fried kobbah samples. L* a* b* is an international standard for color measurement adopted by the Commission Internationale d’Eclairage (CIE) in 1976. L* is the lightness component, which ranges from 0 to 100 and parameters a* (from green to red) and b* (from blue to yellow) are the two chromatic components, which range from −120 to 120 [46]. Total color difference to the control sample (ΔE) indicates the magnitude of color difference between coated kobbah and uncoated control kobbah and it was obtained by the following equation [33,45]:

(1) $$\Delta E=\sqrt{{\left(L*-L’*\right)}^{2}+{\left(a*-a’*\right)}^{2}+\left(b*-b’*\right)²}$$

where L’*, a’* and b’* are the parameters of treated kobbah and L*, a* and b* the ones of the control (uncoated fried kobbah).

The browning index (BI) allowed to define the overall changes in browning color [33,47]. BI of the fried kobbah was calculated by the following equation:

(2) $$\mathrm{BI}=\frac{100\;\left(x-0.31\right)}{0.17}$$

where:

(3) $$X=\frac{\left(a*\;+1.75\;L*\right)}{\left(5.645\;L*\;+\;a*\;-3.012b*\right)}$$

### 2.10. Sodium Dodecyl Sulfate Polyacrylamide Gel Electrophoresis (SDS-PAGE) and In Vitro Digestion

SDS-PAGE, performed as described in Lemmli [48], was carried out at a constant voltage (80 V for 2–3 h). The protein bands were stained with Coomassie Brilliant Blue R250 (Bio-Rad, Segrate, Milan, Italy). Bio-Rad Precision Protein Standards were used as molecular weight markers.

GPF-based FFSs and fried kobbah either treated or not by TGase (33 U/g protein) reinforced or not by MSN, were subjected to in vitro gastric digestion (IVD) by using an adult model [49,50,51]. Then, 100 mg of each sample was incubated with 4 mL of simulated salivary fluid (SSF, 150 mM of NaCl, 3 mM of urea, pH 6.9) containing 75 U of amylase enzyme/g protein for 5 min at 37 °C at 170 rpm [35]. The amylase activity was stopped by adjusting the pH at 2.5. Afterwards, the samples were subjected to IVD as described by Giosafatto et al. [49] and Al-Asmar et al. [35], with some modifications. Briefly, 100 µL of simulated gastric fluid (SGF, 0.15 M of NaCl, pH 2.5) were placed in 1.5 mL microcentrifuge tubes and added to 100 µL of oral phase and then incubated at 37 °C. Thereafter, 50 µL of pepsin (0.1 mg/mL dissolved in SGF) were added to initiate the digestion. At intervals of 1, 2, 5, 10, 20, 40 and 60 min, 40 µL of the 0.5 M of ammonium bicarbonate (NH_4_HCO_3_) were added to each vial to stop the pepsin reaction. The control was set up by incubating the sample for 60 min without the protease. The samples were then analyzed by SDS-PAGE (12%) procedure described above.

### 2.11. Statistical Analysis

The statistical analysis was performed by means of JMP software 10.0 (SAS Institute, Cary, NC, USA), Two-way ANOVA and the *t*-student test for mean comparisons were used. Differences were considered significant at *p* *<* 0.05

## 3. Results and Discussion

### 3.1. Chitosan Nanoparticles, Mesoporous Silica Nanoparticles and Film Forming Solutions Characterization

**Figure 1:**
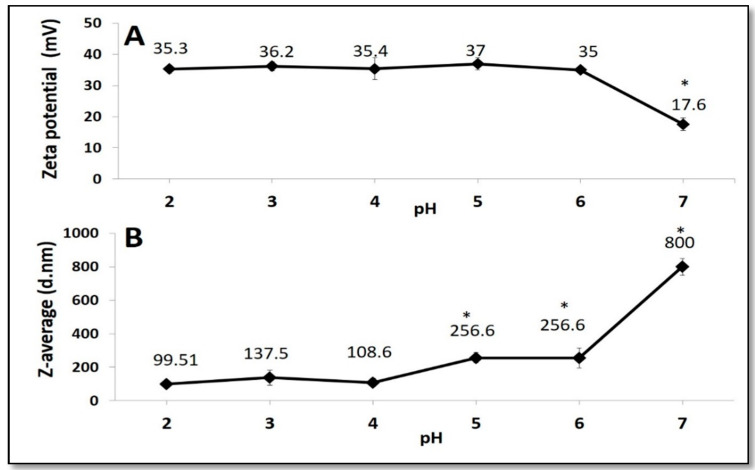
pH effect on Zeta potential (Panel **A**) and Z-average (Panel **B**) of 1 mg/mL chitosan nanoparticles (CH–NPs). Values marked with * were significantly different respect to the value at pH 2.0.

Zeta potential is an important value to indicate the stability of solutions. Moreover, Z-average shows the size of the particles. Figure 1 shows Zeta potential (panel A) and Z-average (panel B) of the CH–NP in the function of pH. The results indicate that Zeta potential of CH–NP was stable at +35 mV started from pH 2.0 to pH 6.0 and decreases to +20 mV when the pH is equal to 7.0. This finding is in accordance with other authors’ results [23,52,53]. The Z-average of CH–NPs at pH 2.0 was around 99.5 d.nm and increased at higher pH to reach 800 d.nm at pH 7.0. The obtained results were in agreement with those from Ali et al. [52], who explained that at pH higher than 6.0 the protonated amino groups start to lose protons and the ionic bonds start decreasing. Thus, the rises of Z-average together with the reduction in Zeta potential at pH 6.0 is because of the particle aggregation at this pH, rather than the additional increase of individual particle size [52,54]. In addition, we synthesized the MSN according to Fernandez-Bats et al. [17], with the very similar Z-average. These authors have analyzed MSN also by TEM and evidenced an average size of 143 ± 26 nm. MSN were used to improve the physio-chemical of PEC and CH films the results reported in Giosafatto et al. [18].

The coating solutions used during this study were also characterized for their stability. Zeta potential and particle size results are reported in Table 1. The results showed that stability was significantly increased after treatment of GPF-based solutions (−13.7 mV) with MSN (−16.8 mV) or TGase (−19.8 mV), also when the enzymatic crosslinking was carried out in of GPF-solutions nanoreinforced either with MSN (−18.4 mV) or CH–NPs (−18.2 mV). However, no significant effect on Zeta potential were found by adding MSN or CH–NPs on PEC FFSs stability. On the contrary, the particle size of GPF solutions was 201.3 d.nm, but this value increased significantly when CH–NP were incorporated with or without TGase. No significant change on the Z-average of FFS after adding MSN on the GPF was observed and these results are similar to those published previously by Fernandez-Bats et al. [17]. Adding TGase as crosslinker to the GPF together with MSN or CH–NP showed a significant increasing on the Z-average of FFSs. In addition, PEC FFS Z-average was (3198 d.nm) and it rises significantly to (3421 d.nm) after the addition of CH–NPs.

### 3.2. Influence on Nanoreinforced and TGase-Crosslinked Hydrocolloid Coating Solutions on Acrylamide Content

**Figure 2:**
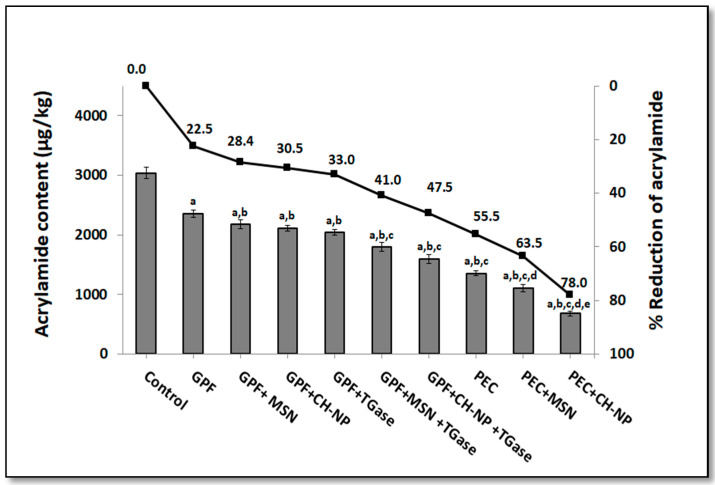
Influence of different hydrocolloid coatings on acrylamide content of fried kobbah (y-axis on the left based on fat-free dry matters (FFDM)) and% acrylamide reduction (y-axis on the right). “Control” represents kobbah sample dipped in distilled water. Columns “a” indicate values significantly different from the control sample; columns “b” indicate values significantly different from grass pea flour (GPF)-coated kobbah; columns “c” indicate values significantly different from GPF + mesoporous silica nanoparticles (MSN)-coated kobbah or GPF + transglutaminase (TGase)-coated kobbah; columns “d” indicate values significantly different from PEC-coated kobbah; columns “e” indicate values significantly different from pectin (PEC) + MSN-coated kobbah. Additional details are reported in the main text.

Kobbah is an ethnic food consumed dispersed among all the world not only in the Arab region. The main aim of this study consisted in studying the effect of the different coating solutions to decrease the ACR content that is formed during frying. The ACR content was performed by RP-HPLC and the results reported in Figure 2. Two main different dipping solutions (GPF and PEC), reinforced by means of two different nanoparticles (1% MSN and 1% CH–NP (*w/w*)) were used to coat the kobbah prior to frying. The GPF was enzymatically crosslinked by means of TGase in the presence or absence of NPs. The control sample was the kobbah dipped into distilled water. Figure 2 shows that control exhibited the highest ACR content reaching a value of 3039.7 µg/kg. On the contrary, all used coating materials were able to significantly reduce ACR content. Kobbah dipped into GPF solution showed about 22.5% reduction in ACR content, while PEC-based coating solution reduced the ACR to 55.5%. The previous work about potato French Fries showed that PEC alone reduced ACR formation about 48% [34]. Coating solutions containing NPs (either MSN or CH–NP) in addition to GPF provoked slightly significant reduction of ACR comparing to the GPF-based coating sample not containing NPs. Higher significant reduction was observed when even MSN or CH–NP were mixed with PEC. The lowest ACR content was detected in the kobbah coated by PEC solutions containing CH–NP. In fact, in these samples the ACR content was 678 µg/kg with the 78.0% ACR reduction in comparison to the control. Recently, Mekawi et al. [31] discovered that the addition of pomegranate peel NP extracts, to the sunflower oil during deep fat frying is responsible for ACR reduction to about (54%) in potato chips. The addition of the enzyme (33U TGase/g GPF protein) into nanoreinforced GPF (even with MSN or CH–NP) reduced the ACR formation significantly (about 41.0% and 47.5%, respectively) in respect to the nanoreinforced GPF prepared without TGase (Figure 2). The obtained data may indicate a potential synergistic effect between NPs and TGase which reduces the Maillard reaction. The ACR recovery was between 93% and 108% (Table 2).

### 3.3. Influence of Nanoreinforced and Crosslinked Hydrocolloid Coating Solutions on Water and Oil Content

**Figure 3:**
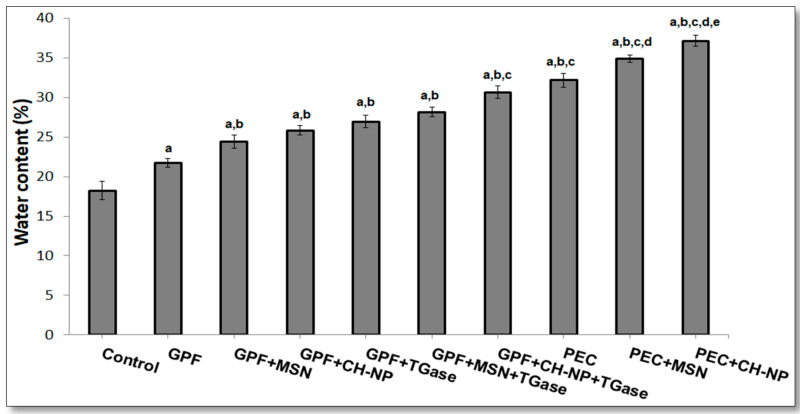
Effect of different hydrocolloid coatings on fried kobbah water content. “Control” represents the kobbah sample dipped in distilled water. Columns “a” indicate values significantly different from the control sample; columns “b” report values significantly different from grass pea flour (GPF)-coated kobbah; columns “c” indicate values significantly different from GPF + mesoporous silica nanoparticles (MSN)-coated kobbah or GPF + transglutaminase (TGase)-coated kobbah; columns “d” indicate values significantly different from pectin (PEC)-coated kobbah; columns “e” indicate values significantly different from PEC + MSN-coated kobbah. Additional details are reported in the main text.

Water content of the kobbah (coated or not) was evaluated and the results reported in Figure 3. The obtained data have shown that the water content significantly increases in kobbah coated with any of the different hydrocolloid solutions used in this research. In fact, the lowest water content was found in the control sample (equal to 18%), while water content in coated kobbah by PEC-based solutions was (32.0%), significantly higher compared to the kobbah coated by GPF-based solutions (21.0%). Nanoreinforcement by using either MSN or CH–NP in both GPF-based or PEC-based solutions, provokes the increasing in water content of the kobbah significantly higher in comparison to samples coated by solutions made of only GPF or PEC. Our findings are supported by Osheba et al. [55], that concluded that CH–NP rise the moisture content of fish fingers up to 52.7%, while the uncoated samples exhibits 34.6% moisture. Regarding the use of TGase, our results prove that the enzyme action in both GPF-based and GPF + NP-based solutions show a significant higher water content respect to the kobbah coated without TGase. Comparable effects were observed by Rossi Marquez et al. [39], where TGase-mediated cross-links are responsible of the reduction of the water evaporation during frying. Moreover, the results demonstrate that the water content of kobbah coated by GPF + CH–NP + TGase is significantly higher compared to the kobbah coated by only GPF + TGase and GPF + MSN + TGase (Figure 3). Recently, Castelo Branco Melo et al. [56] found out that CH–NP led the delaying of the ripening process of the grapes as evident from the decreased weight loss, soluble solids and increased moisture retention.

**Figure 4:**
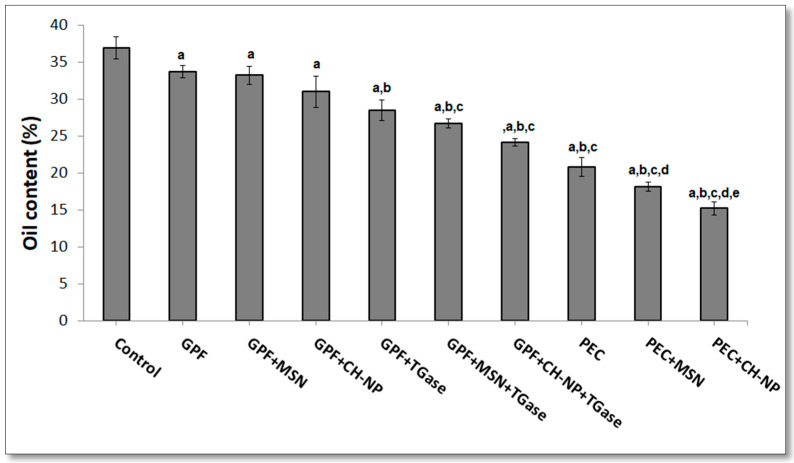
Influence of different hydrocolloid coatings on oil content of fried kobbah. Columns “a” indicate values significantly different from the control sample; columns “b” report values significantly different from grass pea flour (GPF)-coated kobbah; columns “c” indicate values significantly different from GPF + mesoporous silica nanoparticles (MSN)-coated kobbah or GPF + transglutaminase (TGase)-coated kobbah; columns “d” indicate values significantly different from pectin (PEC)-coated kobbah; columns “e” indicate values significantly different from PEC + MSN-coated kobbah. Additional details are reported in the main text.

One of the main health problems is the highest oil content of fried foods. Several studies concluded that coating the fried foods before frying by hydrocolloids materials reduced the oil uptake during frying [39,57]. Figure 4 shows the oil content of kobbah just dipped into water (and used as control) or the ones coated with different solutions. Coating significantly reduces the oil content in comparison to the control, which shows the highest oil content (36.9%), whereas the lowest value was obtained in the fried kobbah coated by PEC + CH–NP (15.2%). There was not any significant difference between the GPF coated kobbah and the kobbah protected by GPF nanoreinforced with MSN or CH–NP. On the other hand, significantly difference in oil content of the fried kobbah were observed between PEC-coated samples and PEC + NP-coated samples. Enzymatically cross-linking of GPF, without and with NPs, demonstrated a significant oil uptake reduction in the coated fried kobbah compared to kobbah coated by GPF or in the presence of NPs (Figure 4). PEC-based coating materials containing NPs (either MSN or CH–NP) induced a significant reduction in oil content of the coated kobbah (18.1% and 15.2%, respectively) compared to kobbah coated with PEC (20.8%). Moreover, using CH–NPs for coating the fish fingers, Osheba et al. [55] have demonstrated a significant reduction of oil uptake which changed from 16.4% in uncoated fish fingers to 4.5% in coated ones.

### 3.4. Influence of Nanoreinforced and Crosslinked Hydrocolloid Coating Solutions on the Kobbah Color

**Figure 5:**
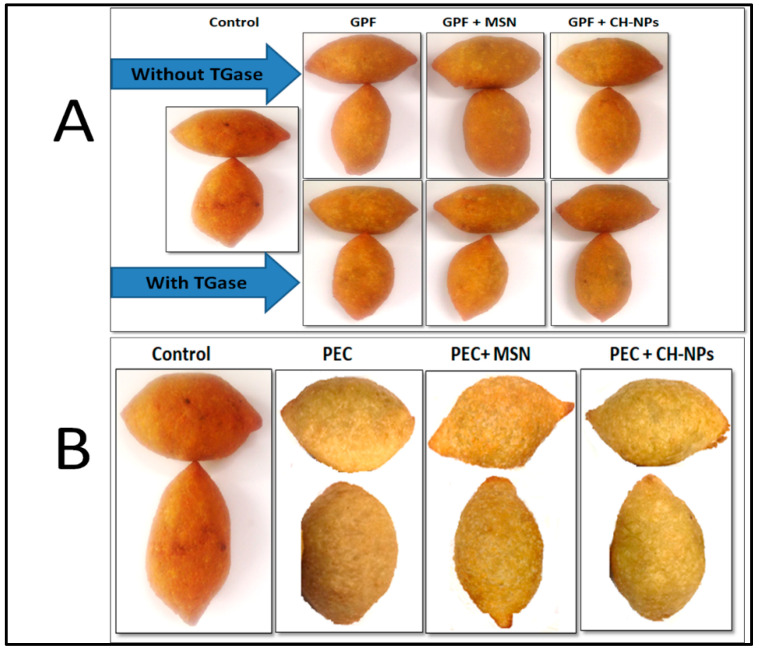
Images of kobbah samples coated by hydrocolloid coatings made of grass pea flour (GPF), GPF + mesoporous silica nanoparticles (MSN), GPF + chitosan nanoparticles (CH–NP), GPF + transglutaminase (TGase); GPF + MSN + TGase and GPF + CH–NP + TGase (Panel **A**); pectin (PEC), PEC + MSN and PEC + CH–NP (Panel **B**). “Control” represents the kobbah sample dipped in distilled water.

Food color is important for the industries, as consumers are highly influenced by this feature. The color is dependent by several processes occurring during food processing [45]. Figure 5 shows the aspect of all the kobbah samples obtained in this study, while the results of color analysis are reported in Table 3, together with L*, a*, b* values and their derivatives, such as total color difference to control (ΔE) and Browning Index (BI).

It was found that color of fried kobbah was influenced by coating which as a consequence could change the color of the final products. The lightness (L*) value showed that the lowest value was in the control samples (49.25 ± 0.68), that is uncoated and the highest value was founded in the kobbah coated by PEC + CH–NP (60.78 ± 1.02) and these results was conformed to Figure 5.

Moreover, kobbah coated with either PEC containing or not MSN or CH–NPs dipping solutions showed significant higher L* value comparing to the kobbah coated with GPF alone or with TGase or nanoparticles. The a* values showed a significant reduction after treated kobbah by different coating solutions the lowest value was in the kobbah coated with PEC + CH–NP (3.32 ± 1.35). GPF containing nanoparticles either with or without TGase showed significant reduction in the a* value comparing to the kobbah coated by only GPF. The b* value showed no significant different between the untreated and the treated kobbah except the coated kobbah by PEC + CH–NP was significant higher comparing to the kobbah coated by GPE.

Total color difference to control (ΔE) showed the highest value was in the kobbah coated by PEC–NP about (12.7 ± 0.98). However, the ΔE of the PEC coating solutions coating nanoparticles was significantly higher comparing to the kobbah coated with GPF solutions. The results indicated that the kobbah coated by the GPF containing CH–NP alone or with TGase was significantly higher than kobbah coated by only GPF (Table 3). In contrast, the highest BI was in the control kobbah (110.09 ± 3.54) and it decreased significantly after coating the kobbah by different coating solutions and the lowest value was detected into the kobbah coated by PEC + CH–NP equal to 79.91 ± 1.72. This result is in correlation with the acrylamide results that indicated that the lowest ACR was in the kobbah coated by PEC–NPs. Jackson and Al-Taher. [58] and EFSA report [30], concluded that the surface color is highly correlated to acrylamide levels, where higher BI means higher ACR content. This demonstrates that the surface browning degree could be an indicator of ACR formation during cooking of kobbah product.

### 3.5. Effect of Nanoreinforced and Crosslinked Hydrocolloid Coating Solutions on the Digestibility of Fried Kobbah

**Figure 6:**
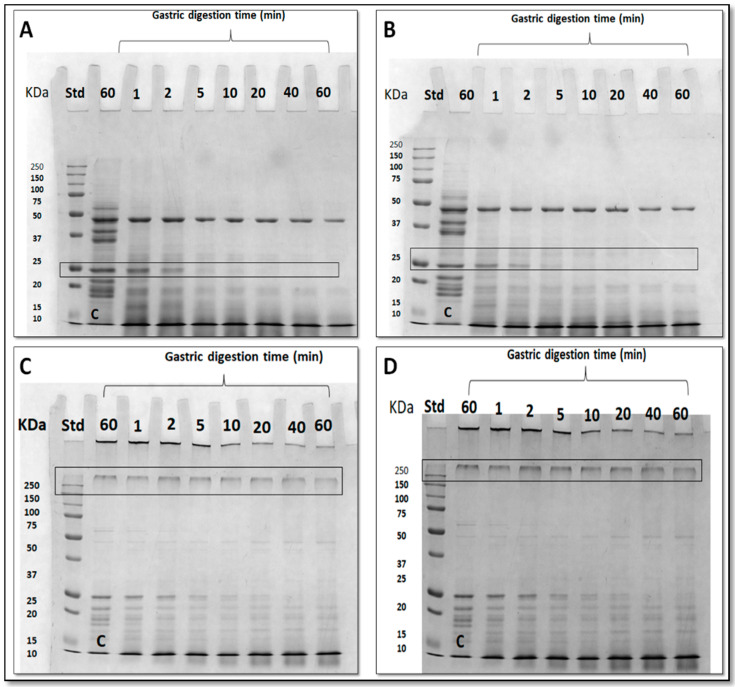
Sodium dodecyl sulfate polyacrylamide gel electrophoresis (SDS-PAGE) of grass pea flour (GPF) film forming solutions (FFSs) after in vitro digestion (IVD) experiments. (Panel **A**): GPF; (Panel **B**): GPF + mesoporous silica nanoparticles (MSN); (Panel **C**): GPF + transglutaminase (TGase 33 *U*/g); (Panel **D**): GPF + MSN + TGase (33 *U*/g). The bands in the rectangle are those chosen for densitometry analysis. C is control sample incubated with simulated gastric fluid (SGF) prepared without pepsin. Std, Molecular mass standards, Bio-Rad.

**Figure 7:**
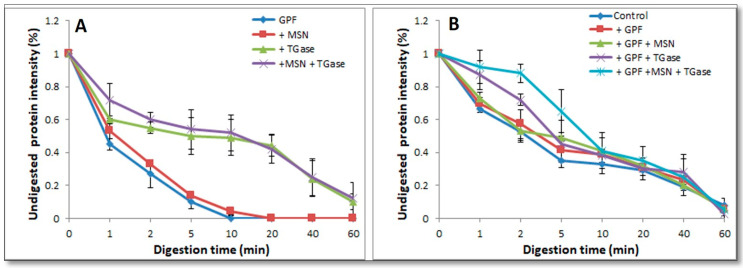
Intensity of the protein framed bands in gels of Figure 6 and Figure 8, obtained after in vitro gastric digestion (IVD). Both grass pea flour (GPF)-based film forming solutions (FFSs) (Panel **A**) and fried kobbah coated with all GPF-based FFSs (Panel **B**), were subjected to densitometry analysis.

In order to verify whether the coating composition could affect digestibility of the fried food, IVD experiments were performed by a protocol set up within the INFOGEST Cost Action [59]. According to INFOGEST protocol, IVD experiments were set up under physiological conditions, followed by SDS-PAGE (12%) analysis as shown in Figure 6. Samples “C” represents the control since such samples were treated with SGF prepared without pepsin. To study the digestibility rate two different kinds of bands were observed: 25 kDa band for samples containing GPF and GPF + MSN and 250 kDa band for samples set up in the presence of the enzyme (GPF + TGase and GPF + MSN + TGase). By visual inspection of the SDS-PAGE patterns of Figure 6, it is possible to assess that MSN do not affect digestibility (comparing Panel B to Panel A and Panel D to Panel C). However, looking at 250 kDa band present in TGase-treated samples (Figure 6, Panels C and D) it is not possible to note significative differences among different samples following pepsin treatment. Thus, densitometry analysis was performed, and the results reported in Figure 7, Panel A. It is possible to note that a significant rate of digestibility of 250 kDa band present in TGase-treated FFS samples, was observed after 10 min pepsin incubation. Similar results were obtained studying digestibility of GPF-based bioplastics crosslinked by means of TGase [8]. Densitometry analysis results of 25 kDa (present in FFS samples not treated with the enzyme) confirmed what was observed by visual inspection, namely an higher digestibility rate already after 1 min pepsin incubation (Figure 7, Panel A). Comparable data were reported by Romano et al. [9].

**Figure 8:**
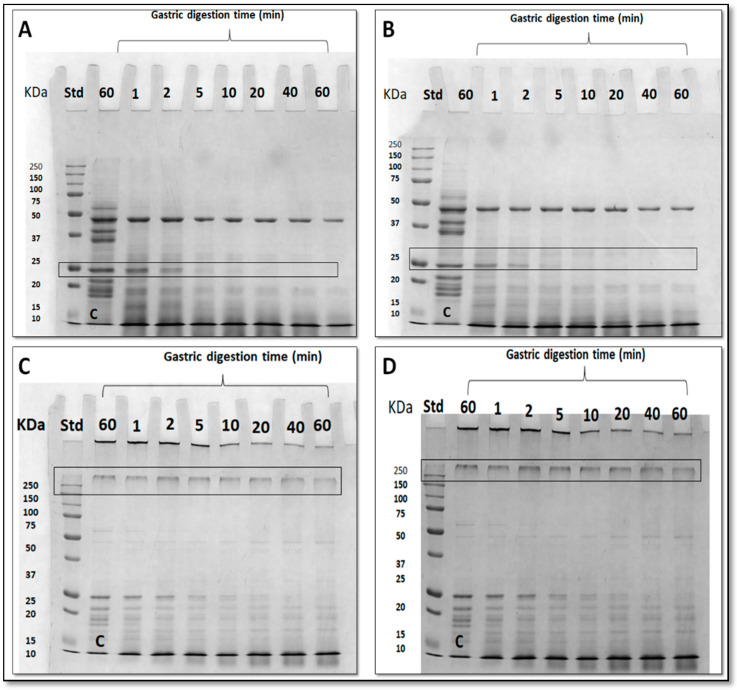
Sodium dodecyl sulfate polyacrylamide gel electrophoresis (SDS-PAGE) of fried kobbah digested by in vitro gastric digestion (IVD) experiments. (Panel **A**): kobbah dipped in water (control); (Panel **B**): kobbah dipped in grass pea flour (GPF); (Panel **C**): kobbah dipped in GPF + mesoporous silica nanoparticles (MSN); (Panel **D**): kobbah dipped in GPF + transglutaminase (TGase 33 *U*/g); (Panel **E**): kobbah dipped in GPF + MSN + TGase (33 *U*/g). The bands in the rectangle are those chosen for densitometry analysis. C is control sample incubated with simulated gastric fluid (SGF) prepared without pepsin. Std, Molecular mass standards, Bio-Rad.

IVD experiments were performed also using kobbah dipped in GPF or GPF containing MSN FFSs-treated (Figure 8). IVD treatment was effective on protein component of kobbah, mainly proteins present in kobbah ingredients (i.e., mostly bulgur flour, beef meat). The ~45 kDa band of samples not treated with TGase was subjected to densitometry analysis, while in enzyme–treated samples the 250 kDa was analyzed.

Densitometry analysis of those bands are observed in Figure 7, Panel B. The digestion seems to be slower in the food coated by protein crosslinked by means of TGase enzyme. However, all the proteins were completely digested by pepsin at the longest incubation time in all different coated kobbah (Figure 7, Panel B).

## 4. Conclusions

Healthier fried kobbah was successfully obtained by dipping method using either GPF or PEC-based solutions. TGase-treated GPF in the presence of nanoparticles was demonstrated to have also important function to reduce ACR formation. The best coating solution that significantly reduced ACR was the one made of PEC nanoreinforced with CH–NP. From the obtained results we conclude that increasing water content inside the fried food by coating is an effective way to mitigate ACR formation and oil content. Reducing the browning index of the fried kobbah is a key indicator to the healthier kobbah. Moreover, the gastric digestion results showed that TGase-mediated modification fairly decreased the rate of digestion in both coating solutions and fried kobbah, even though protein component was completely digested at the end of the longest incubation time.

## Figures and Tables

**Table 1:** Effect of 1% mesoporous silica nanoparticles (MSN) or 1% chitosan nanoparticles (CH–NPs) on Zeta potential and Z-average on either grass pea flour (GPF)-based (without or with transglutaminase (TGase) (33 U/g protein)) or pectin (PEC)-based film forming solutions at pH 6.

FFSs	Zeta Potential (mV)	Z-Average (d.nm)
GPF	−13.7 ± 0.6	201.3 ± 11.1
GPF + MSN	−16.8 ± 0.9 ^a^	191.4 ± 14.2
GPF + CH–NP	−14.1 ± 0.8 ^b^	385.6 ± 28.2 ^a,b^
GPF + TGase	−19.8 ± 1.2 ^a,b^	240.9 ± 14.4 ^a,b^
GPF + MSN + TGase	−18.4 ± 0.5 ^a^	333.0 ± 22.3 ^a,b,c^
GPF + CH–NP + TGase	−18.2 ± 0.9 ^a^	507.7 ± 18.9 ^a,b,c,d^
PEC	−33.7 ± 2.1	3198 ± 79
PEC + MSN	−31.8 ± 2.9	3110 ± 77
PEC + CH–NP	−32.4 ± 3.2	3421 ± 63 *

The value significantly different from GPF FFSs are indicated by “a”, the value indicated by “b” were significantly different from GPF + MSN film forming solution (FFS), whereas the value indicated by “c” were significantly different from GPF + TGase FFS, the value indicated by “d” was significantly different from GPF + MSN + TGase FFSs, the value indicated by “*” was significantly different respect to the PEC and PEC + MSN FFSs. Data represent the average values of three repetitions using (2-way ANOVA, *p* < 0.05 for mean comparison). Additional details are reported in the main text.

**Table 2:** Acrylamide content (ACR) recovery in all kobbah samples (in each sample 150 μg/L of ACR standard were used).

Kobbah Types	ACR Content in Spiked Sample (µg/Kg)	Recovery (%)
Control	3186 ± 61	98
Dipped in GPF	2511 ± 135 ^a^	103
Dipped in GPF + MSN	2329 ± 103 ^a,b^	101
Dipped in GPF + CH–NP	2255 ± 51 ^a,b^	96
Dipped in GPF + TGase	2186 ± 48 ^a,b^	98
Dipped in GPF + MSN + TGase	1934 ± 70 ^a,b,c^	93
Dipped in GPF + CH–NP + TGase	1744 ± 49 ^a,b,c^	99
Dipped in PEC	1495 ± 39 ^a,b,c^	95
Dipped in PEC + MSN	1250 ± 50 ^a,b,c,d^	95
Dipped in PEC + CH–NP	841 ± 37 ^a,b,c,d,e^	108

Values significantly different from those obtained for the controls are indicated by “a”, the value signed with “b” were significantly different from kobbah coated only by grass pea flour (GPF), whereas the value indicated by “c” were significantly different from kobbah coated with GPF in the presence of nanoparticles or TGase alone, the value indicated by “d” were significantly different from kobbah coated only by pectin (PEC) and the value indicated by “e” was significantly different respect to the kobbah coated by PEC + mesoporous silica nanoparticles (MSN). Data reported are the average values of three repetitions using (2-way ANOVA, *p* < 0.05 for mean comparison). Additional details are reported in the main text.

**Table 3:** Color properties of fried kobbah coated with different hydrocolloid-based solutions.

Kobbah Types	L*	a*	b*	ΔE	BI
Control	49.25 ± 0.68	8.28 ± 0.19	31.96 ± 0.76	0.0 ± 0.00	110.09 ± 3.54
Dipped in GPF	51.04 ± 0.34 *	7.77 ± 0.12	31.26 ± 0.57	2.02 ± 0.46 *	100.80 ± 2.99 *
Dipped in GPF + MSN	52.08 ± 1.01 *	6.69 ± 0.33 *^,a^	33.14 ± 0.18	3.48 ± 0.93 *	104.20 ± 3.36
Dipped in GPF CH–NP	53.23 ± 0.97 *^,a^	5.96 ± 0.16 *^,a^	31.88 ± 1.62	4.82 ± 0.76 *^,a^	95.00 ± 8.61 *
Dipped in GPF + TGase	54.93 ± 1.11 *^,a^	6.10 ± 0.82 *^,a^	33.01 ± 0.54	6.20 ± 1.30 *^,a^	95.16 ± 4.36 *^,a^
Dipped in GPF + MSN + TGase	55.35 ± 1.08 *^,a^	5.97 ± 0.31 *^,a^	31.55 ± 1.80	6.70 ± 1.10 *^,a^	88.68 ± 8.02 *^,a^
Dipped in GPF + CH–NP + TGase	55.70 ± 1.00 *^,a^	5.81 ± 0.42 *^,a^	32.06 ± 1.29	7.01 ± 0.84 *^,a^	89.44 ± 6.35 *^,a^
Dipped in PEC	56.56 ± 0.69 *^,a,b^	5.44 ± 0.31 *^,a^	32.58 ± 0.36	7.87 ± 0.75 *^,a,b^	88.75 ± 2.02 *^,a^
Dipped in PEC + MSN	58.03 ± 0.77 *^,a,b^	5.01 ± 0.25 *^,a^	32.99 ± 0.38	9.36 ± 0.77 *^,a,b,c^	86.78 ± 3.18 *^,a^
Dipped in PEC + CH–NP	60.78 ± 1.02 *^,a,b,c^	3.32 ± 1.35 *^,a,c^	33.34 ± 1.00 ^a^	12.70 ± 0.98 *^,a,b,c^	79.91 ± 1.72 *^,a,c^

Columns significantly different from those obtained by analyzing the control are indicated by “*”, the columns indicated by “^a^” were significantly different from kobbah coated only by grass pea flour (GPF), whereas the columns with “^b^” were significantly different from kobbah coated by GPF with transglutaminase (TGase) alone, the columns indicated by “^c^” were significantly different from kobbah coated only by pectin (PEC) or PEC in presence of mesoporous silica nanoparticles (MSN). The results represent the average values of three repetitions using (2-way ANOVA, *p* < 0.05 for mean comparison). Additional details are reported in the main text.
